# Pilot-Scale Production and Thermostability Improvement of the M23 Protease Pseudoalterin from the Deep Sea Bacterium *Pseudoalteromonas* sp. CF6-2

**DOI:** 10.3390/molecules21111567

**Published:** 2016-11-17

**Authors:** Jie Yang, Yang Yu, Bai-Lu Tang, Shuai Zhong, Mei Shi, Bin-Bin Xie, Xi-Ying Zhang, Bai-Cheng Zhou, Yu-Zhong Zhang, Xiu-Lan Chen

**Affiliations:** 1 State Key Laboratory of Microbial Technology, Shandong University, Jinan 250100, China; yangjie199102@163.com (J.Y.); yuyang15643177899@163.com (Y.Y.); tangbailu@yeah.net (B.-L.T.); servetonentsh@hotmail.com (S.Z.); clubstone@sdu.edu.cn (M.S.); xbb@sdu.edu.cn (B.-B.X.); zhangxiying@sdu.edu.cn (X.-Y.Z.); zhangyz@sdu.edu.cn (Y.-Z.Z.); 2 Marine Biotechnology Research Center, Shandong University, Jinan 250100, China; bczhou@qdio.ac.cn; 3 Laboratory for Marine Biology and Biotechnology, Qingdao National Laboratory for Marine Science and Technology, Qingdao 266000, China

**Keywords:** marine bacteria, M23 proteases, fermentation, optimization, stability, cold-adapted enzymes

## Abstract

Pseudoalterin is the most abundant protease secreted by the marine sedimental bacterium *Pseudoalteromonas* sp. CF6-2 and is a novel cold-adapted metalloprotease of the M23 family. Proteases of the M23 family have high activity towards peptidoglycan and elastin, suggesting their promising biomedical and biotechnological potentials. To lower the fermentive cost and improve the pseudoalterin production of CF6-2, we optimized the fermentation medium by using single factor experiments, added 0.5% sucrose as a carbon source, and lowered the usage of artery powder from 1.2% to 0.6%. In the optimized medium, pseudoalterin production reached 161.15 ± 3.08 U/mL, 61% greater than that before optimization. We further conducted a small-scale fermentation experiment in a 5-L fermenter and a pilot-scale fermentation experiment in a 50-L fermenter. Pseudoalterin production during pilot-scale fermentation reached 103.48 ± 8.64 U/mL, 77% greater than that before the medium was optimized. In addition, through single factor experiments and orthogonal tests, we developed a compound stabilizer for pseudoalterin, using medically safe sugars and polyols. This stabilizer showed a significant protective effect for pseudoalterin against enzymatic thermal denaturation. These results lay a solid foundation for the industrial production of pseudoalterin and the development of its biomedical and biotechnological potentials.

## 1. Introduction

Due to the yearly deposition of particulate organic nitrogen from seawater to deep-sea sediment [1], protease-producing bacteria thrive in deep-sea sediments [2,3], which provides an ample resource to exploit novel proteases with biotechnological potential. Novel proteases from deep-sea sedimental bacteria have been reported, such as the S8 collagenolytic protease MCP-01 from *Pseudoalteromonas* sp. SM9913 [4,5], the S8 collagenolytic protease myroicolsin from *Myroides profundi* D25 [6], the M12 elastic protease myroilysin from *Myroides profundi* D25 [7], the M13 metallopeptidase PepS from *Shewanella* sp. E525-6 [8], the alkaline protease from *Alkaliphilic actinomycetes* MA1-1 [9], the thermolysin-like protease HSPA from *Halobacillus* sp. SCSIO 20089 [10] and the M23 metalloprotease pseudoalterin from *Pseudoalteromonas* sp. CF6-2 [11].

*Pseudoalteromonas* sp. CF6-2 (hereafter CF6-2) is a protease-producing bacterium isolated from a South China Sea sediment [2]. Pseudoalterin, the most abundant protease secreted by CF6-2, is a novel metalloprotease from the M23 family. Proteases in the M23 family are known to specifically cleave the cross-linking pentaglycine bridges in peptidoglycans in bacterial cell wall [12,13] and the glycyl bonds in elastin [14,15]. Lysostaphin and staphylolysin in the M23 family were reported to lyse methicillin-resistance *Staphylococcus aureus* (MRSA) and vancomycin-intermediate *S. aureus* (VISA) [16] and to be effective in treating experimental *S. aureus* keratitis in rabbits [17,18,19]. These studies suggest that the proteases from the M23 family may have promising therapeutic values for the treatment of antibiotic-resistant infections in humans. Compared to lysostaphin and staphylolysin, pseudoalterin has a higher elastolytic activity as a result of a broader specificity. It cleaves both glycyl bonds in the hydrophobic domains and peptide bonds in the hydrophilic domains of elastin [11]. This distinct elastolytic property suggests that pseudoalterin may possess the ability to lyse bacterial cell walls and therefore has potential in the biomedical field. However, the production of pseudoalterin in CF6-2 is low, and it is a cold-adapted enzyme with low thermostability. The half-life of pseudoalterin activity was only 14.13 min at 35 °C [11]. Therefore, it is necessary to improve the production and thermostability of pseudoalterin for potential development in biomedical and biotechnological fields.

Heterologous expression, especially in *Escherichia coli*, is a conventional method to improve enzyme production [20,21]. Protein-engineering approaches, such as random mutagenesis, directed evolution and site-directed mutagenesis, have been commonly used to improve the thermostability of cold-adapted enzymes [22,23]. However, because the M23 proteases are not autoprocessed during maturation [24], it is almost impossible to obtain the mature form of M23 proteases through heterologous expression. Therefore, it is worthy to improve the production of these enzymes in their wild strains by optimizing the fermentation conditions and to improve their thermostability by the addition of chemical stabilizers. Pseudoalterin is an inducible enzyme that utilizes elastin as an inducer [11]. We previously developed a fermentation medium with artery powder in place of elastin as an inducer for the production of pseudoalterin in CF6-2 and optimized flask fermentation conditions by response surface methodology to lower fermentation costs and to improve enzyme production [25]. In this study, we further optimized the fermentation medium by lowering the content of artery powder and increasing the carbon source content in the medium. With the optimized medium, small-scale fermentation in a 5-L fermenter and pilot-scale fermentation in a 50-L fermenter were conducted. Moreover, a compound stabilizer, which can significantly improve the thermostability of pseudoalterin and other enzymes, was developed by single factor experiments and an orthogonal test.

## 2. Results

### 2.1. Optimization of the Fermentation Medium by Increasing the Content of the Carbon Source and Lowering the Usage of Artery Powder

The flask fermentation medium previously developed for pseudoalterin production of CF6-2 contained 1.2% (weight/volume, *w*/*v*) artery powder, 0.3% (*w*/*v*) yeast extract, 0.5 mM Na_2_HPO_4_, 0.5 mM CaCl_2_ and artificial sea water (pH 8.5) [25]. This medium is termed the basal medium in this study. We found that the artery powder served not only as an inducer for pseudoalterin production but also as a main carbon source for CF6-2 growth in the basal medium. Because bovine artery powder is more expensive than common carbon sources, such as corn powder, glucose and sucrose, we sought to replace a part of the artery powder content with common carbon sources in the CF6-2 fermentation medium.

**Figure 1:**
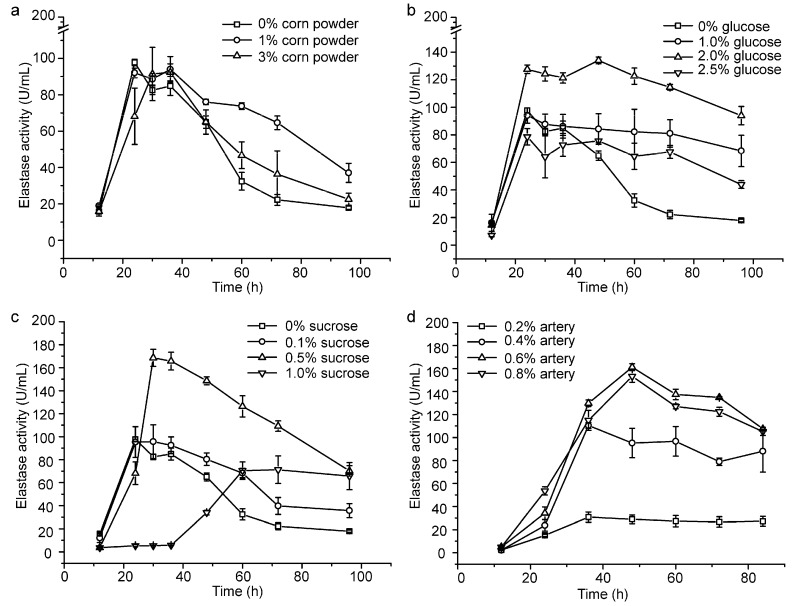
Optimization of the fermentation medium of CF6-2 for pseudoalterin production. (**a**) Effect of the corn powder content on pseudoalterin production; (**b**) Effect of the glucose content on pseudoalterin production; (**c**) Effect of the sucrose content on pseudoalterin production; (**d**) Effect of the artery powder content on pseudoalterin production in the basal medium, containing 0.5% sucrose as a carbon source. The graphs show data from treatments performed in triplicate (mean ± S.D.).

We investigated the effects of corn powder, glucose and sucrose on the production of pseudoalterin by adding them into the basal medium. Because pseudoalterin has a high elastase activity and is the most abundant protease secreted by CF6-2 [11], we used the elastase activity in the CF6-2 culture to represent the production of pseudoalterin in the culture. While an increase in the content of corn powder from 0% to 3% caused only a few changes in pseudoalterin production (Figure 1a), the contents of glucose and sucrose in the medium had a significant influence on pseudoalterin production (Figure 1). When the glucose content was set as a single factor variable in the basal medium, pseudoalterin production was greatest (134.10 ± 2.42 U/mL) in the medium containing 2% glucose (Figure 1b). With sucrose as a single factor variable in the medium, pseudoalterin production peaked (168.59 ± 7.42 U/mL) at 0.5% sucrose (Figure 1c). These results indicate that sucrose as a carbon source is better than glucose or corn powder for pseudoalterin production. Therefore, 0.5% sucrose was used as the carbon source in the following experiment to lower the usage of artery powder.

To reduce the usage of artery powder in the medium, flask fermentation of CF6-2 was performed with artery powder (0.2%~0.8%) as the single factor variable in the basal medium containing 0.5% sucrose. Pseudoalterin production was highest (161.15 ± 3.08 U/mL) in the medium containing 0.6% artery powder (Figure 1d). Therefore, with the addition of 0.5% sucrose as carbon source in the medium, the content of artery powder was reduced from 1.2% to 0.6%, and pseudoalterin production reached 161% of that in the basal medium (100.02 ± 9.0 U/mL) [25].

Based on the results from the above single factor experiments, we obtained an optimized flask fermentation medium for CF6-2, which contained 0.6% (*w*/*v*) artery powder content, 0.5% (*w*/*v*) sucrose, 0.3% (*w*/*v*) yeast extract, 0.5 mM NaH_2_PO_4_, 0.5 mM CaCl_2_, and artificial sea water (pH 8.5). Compared to the basal medium, CF6-2 fermentation in this optimized medium led to a reduction in productive cost and an increase in pseudoalterin production.

### 2.2. Small-Scale Fermentation of CF6-2 for Pseudoalterin Production

**Figure 2:**
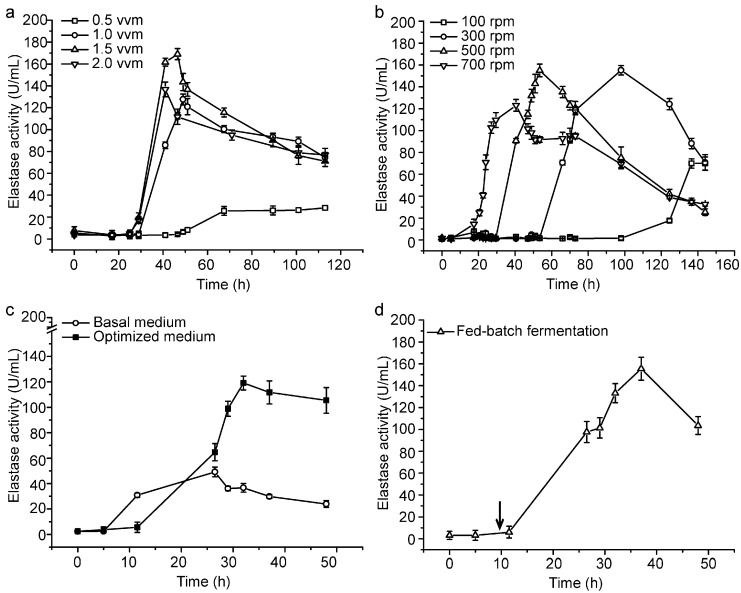
Small-scale fermentation of CF6-2 for pseudoalterin production. (**a**,**b**) Effects of the aeration rate (**a**) and the rotation speed (**b**) on pseudoalterin production by CF6-2. CF6-2 was cultured in the optimized medium in a mini-in parallel fermenter system; (**c**) The pseudoalterin production by CF6-2 in the optimized medium and in the basal medium in a 5-L fermenter; (**d**) The pseudoalterin production of CF6-2 under the fed-batch fermentation process in a 5-L fermenter. The arrow indicates the time when a 0.5-L feeding medium was supplemented. The graphs show data from treatments performed in triplicate (mean ± S.D.).

With the optimized medium, we further conducted a small- and pilot-scale fermentation experiment for CF6-2 to lay the foundation for industrial production of pseudoalterin. Firstly, we determined the optimal aeration rate and stirring speed for the production of pseudoalterin in a mini-in parallel fermenter system. When CF6-2 was cultured at 0.5 vessel volume per minute (vvm), 1.0 vvm, 1.5 vvm and 2.0 vvm, respectively, the broth at 1.5 vvm showed the highest elastase activity (Figure 2a). When CF6-2 was cultured at 100 revolutions per minute (rpm), 300 rpm, 500 rpm and 700 rpm, respectively, the broth at 500 rpm showed the highest elastase activity (Figure 2b). Therefore, 1.5 vvm and 500 rpm were chosen for small-scale fermentation in a 5.0-L fermenter. In the small-scale fermentation, with a 1% inoculation and 80% loading volume, CF6-2 was cultured at 20 °C with an initial aeration rate of 0.5 vvm and initial stirring speed of 300 rpm. After a 5-h culturation, when strain growth entered the logarithmic growth phase, the aeration rate was adjusted to 1.5 vvm, and the stirring speed was adjusted to 500 rpm, which were maintained until the end of fermentation. The pseudoalterin production of CF6-2 reached 119.11 ± 5.43 U/mL, which was 142% higher than that (49.13 ± 3.19 U/mL) in the basal medium using this fermentation technique (Figure 2c).

Based on the small-scale fermentation technique, we investigated fed-batch fermentation to further improve the production of pseudoalterin. At the 9th hour of fermentation, 0.5 L of the feeding medium, which contained 1% (*w*/*v*) artery powder, 0.2% (*w*/*v*) yeast extract, 0.33% (*w*/*v*) sucrose, 0.5 mM Na_2_HPO_4_, 0.5 mM CaCl_2_ and artificial sea water (pH 8.5), was supplemented. After the medium was fed, pseudoalterin production of CF6-2 reached 155.57 ± 10.45 U/mL (Figure 2d). Therefore, the addition of feeding medium could further improve the pseudoalterin production of CF6-2.

### 2.3. Pilot-Scale Fermentation of CF6-2 for Pseudoalterin Production

**Figure 3:**
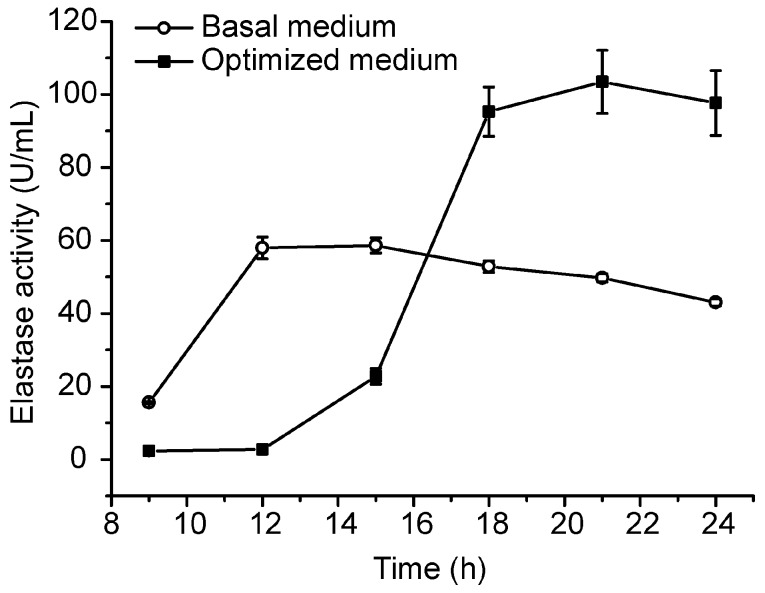
Comparison of the pseudoalterin production by CF6-2 in the optimized medium and in the basal medium in a pilot-scale fermentation. The graph shows data from treatments performed in triplicate (mean ± S.D.).

Based on the small-scale fermentation technique, we conducted a pilot-scale fermentation of CF6-2 in a 50-L fermenter. In the pilot-scale fermentation, 35 L of the optimized medium and 3 mL of the antifoaming agent were loaded in the 50-L fermenter, and 1% volume of CF6-2 seed culture was inoculated into the medium, which was then cultured at 20 °C with an aeration rate of 0.5 vvm and a stirring speed of 300 rpm. After 5 h culturation, the aeration rate was adjusted to 1.5 vvm and the stirring speed to 500 rpm to increase the dissolved oxygen in the culture to meet the increase of oxygen consumption. At the 9th hour of culturation, 5 L of the feeding medium was added in the fermenter. Under this fermentation technique, the pseudoalterin production of CF6-2 reached 103.48 ± 8.64 U/mL, which was 77% higher than that (58.62 ± 2.09 U/mL) in the basal medium under the same pilot fermentation conditions (Figure 3).

### 2.4. Effects of Sugars and Polyols on the Thermostability of Pseudoalterin

**Figure 4:**
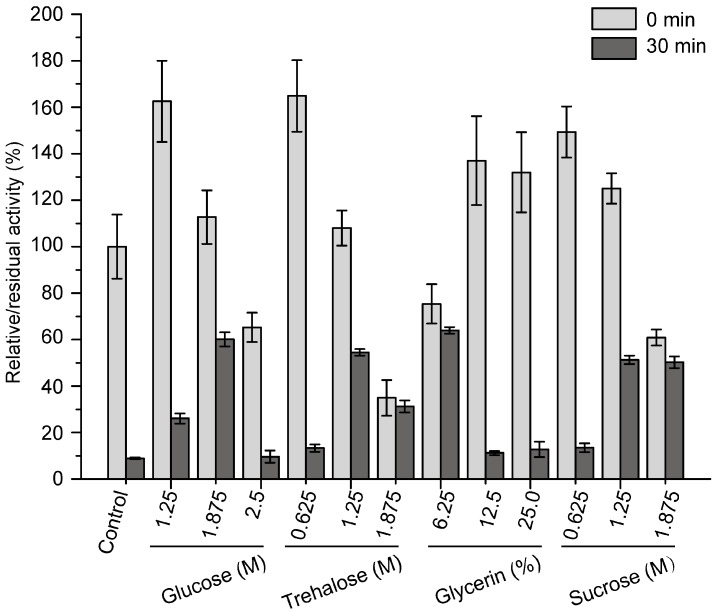
Effects of sugars and polyols on the activity and thermostability of pseudoalterin. The enzyme solution was mixed with a varying concentrations of glucose, trehalose, glycerin or sucrose, and incubated at 37 °C for 30 min. The enzyme solution, mixed with 50 mM Tris-HCl (pH 9.0), served as the control. The elastase activity of the mixture before and after incubation was measured. The activity of the enzyme solution mixed with 50 mM Tris-HCl (pH 9.0) before incubation was taken as 100%. Experiments were performed in triplicate, and error bars indicate standard deviation.

A previous study showed that pseudoalterin is a cold-adapted enzyme with a low thermostability [11]. Because the low thermostability of pseudoalterin is a hindrance for its storage and application, it is necessary to improve its thermostability. Pseudoalterin could not be expressed in its mature form by *E. coli*, which makes it difficult to improve its thermostability by protein engineering. Thus, we developed a compound stabilizer to improve its thermostability. We purified pseudoalterin from the culture supernatant of CF6-2 by using a DEAE-Sepharose Fast Flow column as previously reported [11]. Considering that pseudoalterin could be developed as a human medicine in the future, medically safe sugars and polyols, including glucose, trehalose, glycerin and sucrose, were chosen to develop the compound stabilizer for pseudoalterin. The effects of glucose, trehalose, glycerin and sucrose on the activity and thermostability of purified pseudoalterin were evaluated. All four compounds improved the activity of pseudoalterin at certain concentrations and showed significant protective effects on pseudoalterin against heat denaturation at 37 °C in comparison to the control (Figure 4). For each of these compounds, 1.875 M glucose, 1.25 M trehalose, 6.25% glycerin and 1.25 M sucrose showed the highest protective effect, which all resulted in approximately 60% retention of the original enzyme activity after a 30-min incubation at 37 °C, approximately 6-fold higher than that of the enzyme in the absence of any of the compounds (Figure 4).

### 2.5. Development of an Effective Compound Stabilizer for Pseudoalterin by an Orthogonal Test

**Figure 5:**
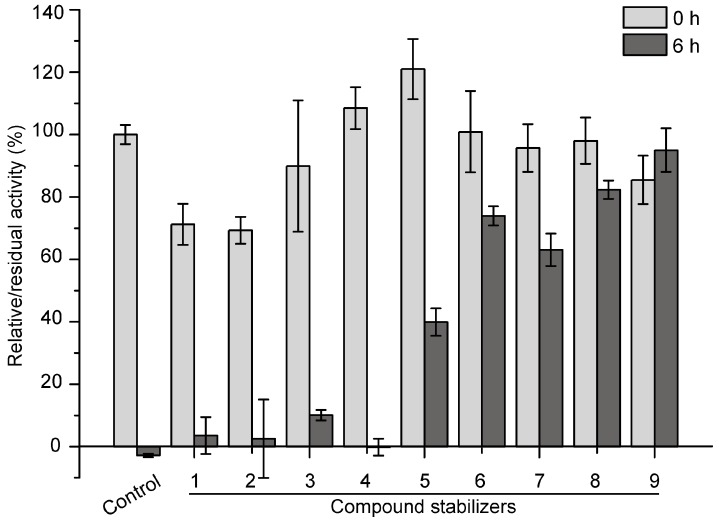
Effects of compound stabilizers on the activity and thermostability of pseudoalterin. The enzyme solution was mixed with each compound stabilizer and incubated at 37 °C for 6 h. The enzyme solution, mixed with 50 mM Tris-HCl (pH 9.0), served as the control. The elastase activity of the mixture before and after incubation was measured. The activity of the enzyme solution mixed with 50 mM Tris-HCl (pH 9.0) before incubation was taken as 100%. Experiments were performed in triplicate, and error bars indicate standard deviation.

Based on the results of single factor experiments shown in Figure 4, we further used an orthogonal test to design compound stabilizers for pseudoalterin. As shown in Table 1 and Table 2, 9 compound stabilizers were designed by an orthogonal test. While these compound stabilizers had few effects on the activity of pseudoalterin, most showed significant protective effects on pseudoalterin against thermal denaturation (Figure 5). Among them, compound stabilizer 9 displayed the highest protective effect. In compound stabilizer 9, pseudoalterin retained 90% of the original enzyme activity after 6 h of incubation at 37 °C. Compound stabilizer 8 showed the second highest protective effect, making pseudoalterin retain 82% of the original enzyme activity after a 6-h incubation at 37 °C. Therefore, compound stabilizer 9 is the most effective stabilizer to improve the thermostability of pseudoalterin, which contained 1.875 M glucose, 1.25 M trehalose, 2.5% glycerin and 0.25 M sucrose in 50 mM Tris-HCl (pH 9.0).

### 2.6. Effect of Compound Stabilizer 9 on the Thermostability of Pseudoalterin as Well as Other Enzymes

**Figure 6:**
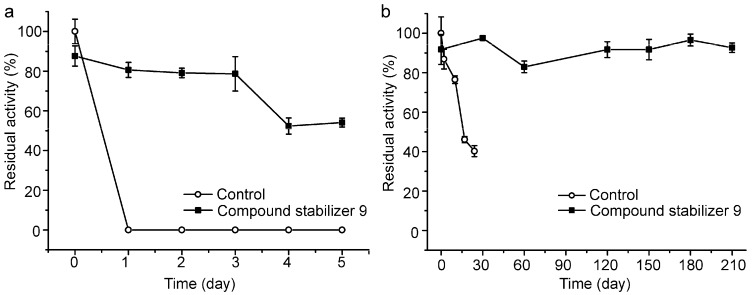
Thermostability of pseudoalterin preserved in compound stabilizer 9 at 37 °C (**a**) and 4 °C (**b**). The enzyme solution was mixed with compound stabilizer 9 and incubated at 37 °C for 5 days and at 4 °C for 210 days. The enzyme solution in 50 mM Tris-HCl (pH 9.0) served as the control. The activity of the enzyme solution in 50 mM Tris-HCl (pH 9.0) before incubation was taken as 100%. Experiments were performed in triplicate, and error bars indicate standard deviation.

We further investigated the long-term effect of compound stabilizer 9 on the thermostability of pseudoalterin incubated at 37 °C and 4 °C. In the presence of compound stabilizer 9, pseudoalterin at 37 °C retained 80% of its original activity after three days and 50% after five days (Figure 6a). At 4 °C, the activity of pseudoalterin preserved in the presence of compound stabilizer 9 remained quite stable after 210 days, but the enzyme preserved in the absence of compound stabilizer 9 lost almost 60% of the activity after 24 days (Figure 6b). These results indicate that compound stabilizer 9 is a good compound stabilizer for the transportation, storage and application of pseudoalterin.

**Figure 7:**
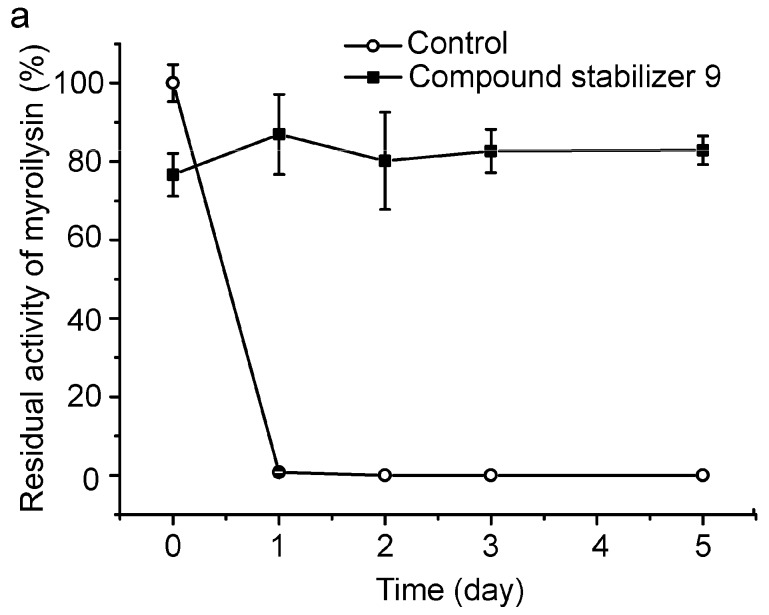

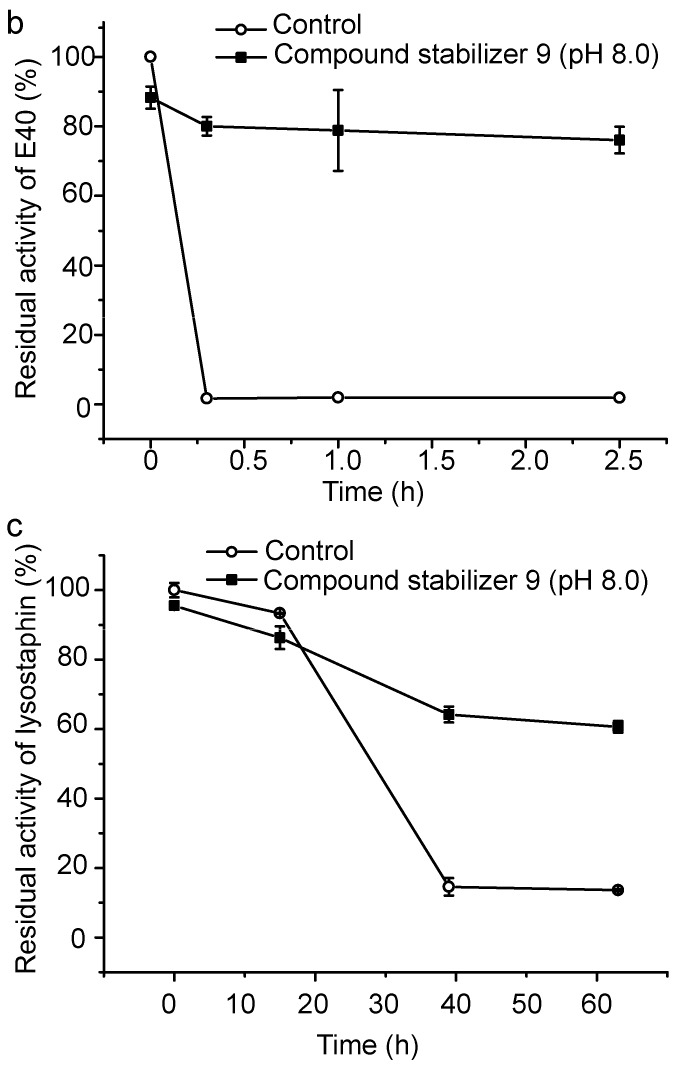
Effect of compound stabilizer 9 on the thermostability of myroilysin (**a**); E40 (**b**); and lysostaphin (**c**). Each enzyme mixed with compound stabilizer 9 was incubated at 37 °C. The activity of the enzyme solution in 50 mM Tris-HCl (pH 9.0) before incubation at 37 °C was taken as 100%. Experiments were performed in triplicate, and error bars indicate standard deviation.

To evaluate its universal use, we further investigated the protective effect of compound stabilizer 9 on three enzymes. Myroilysin is an M12 elastase secreted by a deep-sea cold-adapted bacterium [7]. E40 is a thermolabile HSL esterase isolated from a marine sedimental metagenomic library [26]. Lysostaphin is an M23 protease produced by *Staphylococcus simulans* [27]. Because myroilysin has the same optimal pH of 9.0 as pseudoalterin [7,11], compound stabilizer 9 (pH 9.0) was directly used to investigate its effect on myroilysin thermostability. As shown in Figure 7a, myroilysin incubated with compound stabilizer 9 at 37 °C retained 80% of its original activity after five days, whereas the control enzyme without compound stabilizer 9 lost all its activity after one day, demonstrating the potent effect of compound stabilizer 9 on improving myroilysin thermostability. Because the optimal pH of E40 and lysostaphin is 8.0 [16,26], we adjusted the pH of compound stabilizer 9 to 8.0 before testing its effect on the thermostability of E40 and lysostaphin. E40 was quite thermolabile, losing all its activity after incubation at 37 °C for only 20 min. In contrast, in the presence of compound stabilizer 9 (pH 8.0), E40 retained almost 80% of its original activity after incubation at 37 °C for 2.5 h (Figure 7b). Similarly, lysostaphin incubated with compound stabilizer 9 (pH 8.0) at 37 °C retained approximately 60% of its original activity after 80 h, approximately 6-fold higher than that of the control enzyme incubated without compound stabilizer 9 (Figure 7c). Altogether, these results show that compound stabilizer 9 can significantly improve enzyme thermostability, which indicates that compound stabilizer 9 can be used as a stabilizer not only for pseudoalterin but also for other thermolabile enzymes.

## 3. Discussion

For the production of microbial enzymes with biotechnological and/or industrial potential, it is important to lower the fermentation cost and improve the yield. Because pseudoalterin, a novel M23 protease from the marine sedimental bacterium *Pseudoalteromonas* sp. CF6-2, has potential biomedical and biotechnological use [11], we lowered its fermentation cost and improved the production [25]. In a previous study, we used artery powder instead of expensive elastin and optimized the flask fermentation conditions of CF6-2, which yielded 100.02 ± 9.0 U/mL pseudoalterin [25]. In this study, we optimized the fermentation medium to further lower the fermentation cost and conducted small- and pilot-scale fermentations.

Through optimization, we added 0.5% sucrose and lowered the content of artery powder from 1.2% to 0.6% in the previously optimized medium (the basal medium). Because sucrose is a cheaper and more available commercial agent than artery powder, which is not commercial, the optimized medium is cheaper and easier to prepare compared to the basal medium. Moreover, pseudoalterin production reached 161.15 ± 3.08 U/mL in the optimized medium, 161% of that in the basal medium [25]. Therefore, the fermentive medium optimization performed in this study is effective to lower the fermentation cost and to improve the production of pseudoalterin of CF6-2. To lay the foundation for industrial production, we conducted small- and pilot-scale fermentations of CF6-2 for pseudoalterin production in the optimized medium. In the small-scale fermentation in a 5-L fermenter, pseudoalterin production reached 119.11 ± 5.43 U/mL, 142% higher than that (49.13 ± 3.19 U/mL) in the basal medium under the same fermentation conditions. In the pilot-scale fermentation in a 50-L fermenter, pseudoalterin production reached 103.48 ± 8.64 U/mL, 177% of that in the basal medium under the same fermentation conditions. Thus, we established a pilot-scale fermentation process of CF6-2 for pseudoalterin production.

Due to adaptation to the permanently cold deep-sea sediment, pseudoalterin is a cold-adapted enzyme with a low thermostability [11]. Cold-adapted enzymes tend to lose their activity at moderate and even low temperatures [27,28,29], which is detrimental for their storage and application. Therefore, it is important to improve the thermostability of pseudoalterin before developing its applications. Because pseudoalterin cannot be actively expressed in *E. coli*, we decided to develop a compound stabilizer to improve its thermostability. It has been reported that sugars and polyols can stabilize proteins against heat denaturation. With the addition of sugars and polyols, the hydrophobic interactions among nonpolar amino acid residues of the enzymes are strengthened, which are vital to maintain the structure of the enzymes. As a result, with the presence of sugars and polyols, enzyme macromolecules tend to be more rigid and therefore more resistant to thermal unfolding [30]. For example, Samborska et al. reported that sucrose, trehalose and glycerin had significant protective effects on α-amylase thermostability [31]. Moreover, compound stabilizers can take full advantage of the benefits of diverse sugars and polyols. The interactions between a compound stabilizer and enzyme are more diverse and complex, which provide a better protective effect on an enzyme than one compound [30,31]. Therefore, we used medically safe sugars and polyols, including glucose, trehalose, glycerin and sucrose, to develop a compound stabilizer to improve the thermostability of pseudoalterin. By single factor experiments and an orthogonal test, we developed an effective compound stabilizer for pseudoalterin, which allowed pseudoalterin to retain 50% of its activity after a five-day incubation at 37 °C and to retain all its activity after seven months at 4 °C. Therefore, this compound stabilizer can improve the thermostability of pseudoalterin significantly. Moreover, this compound stabilizer also improved the thermostability of multiple other enzymes, indicating that it may be used as a universal stabilizer for thermolabile enzymes.

In summary, using findings from a previous study, the fermentation medium for pseudoalterin production of CF6-2 was further optimized, and a pilot-scale fermentation process was set up. The thermostability of pseudoalterin was significantly improved by developing an effective compound stabilizer. The results in this study lay a solid foundation for developing the industrial production and applications for pseudoalterin.

## 4. Materials and Methods

### 4.1. Strains and Media

*Pseudoalteromonas* sp. CF6-2 was isolated from the sediment at a 2441-m water depth from the South China Sea at site 119°30.060′ E, 22°0.316′ N during the 2007 South China Sea Open Cruise by R/V Shiyan 3 [2] and was deposited in the China Center for Type Culture Collection (Wuhan, China) under the accession no. CCTCC M2010189. Strain CF6-2 was cultured at 20 °C for 24 h on a plate containing (*w*/*v*) 1% peptone, 0.5% yeast extract, 1.5% agar and artificial sea water (pH 8.0) and then stored at 4 °C for short-term use. The basal medium previously developed for pseudoalterin production of CF6-2 contained 1.2% (*w*/*v*) artery powder, 0.3% (*w*/*v*) yeast extract, 0.5 mM Na_2_HPO_4_, 0.5 mM CaCl_2_ and artificial sea water, with an initial pH of 8.5 and a culture volume of 50 mL/500 mL [25]. All chemical reagents used in this study were of analytical reagent grade. Bovine artery was purchased from a local abattoir and processed into artery powder as previously described [25]. Antifoaming agent (polyoxypropylene-oxyethylene polyol ether) was kindly provided by the Chemical Factory of Shandong Normal University, China.

### 4.2. Inoculum Preparation and Flask Fermentation

To prepare the inoculum, CF6-2 was cultured at 20 °C and 180 rpm for 12 h in a marine broth medium containing 1% (*w*/*v*) peptone, 0.5% (*w*/*v*) yeast extract and artificial sea water (pH 8.0). For flask fermentation, a 1% (volume/volume, *v*/*v*) inoculum with the absorption at 600 nm of approximately 1.5 was inoculated into 50 mL of the basal medium in a 500-mL Erlenmeyer flask, which was then incubated at 20 °C with a stirring speed of 180 rpm for 96 h.

### 4.3. Enzyme Assay

The fermentation broth of CF6-2 was centrifuged at 11,000 rpm at 4 °C for 15 min, and the supernatant was collected for the enzyme assay. After being appropriately diluted with 50 mM Tris-HCl (pH 9.0), the elastase activity of the supernatant was assayed with a previously described method [7]. Briefly, 0.25 mL of the enzyme solution was incubated with 5 mg of elastinorcein at 25 °C for 60 min with continuous shaking. The supernatant was collected after centrifugation, and its absorption at 590 nm was measured. One unit of enzyme activity was defined as the amount of enzyme required to cause an increase of 0.01 unit of absorbance at 590 nm per min. The activity of myroilysin, E40 and lysostaphin was assayed as previously described [7,16,26].

### 4.4. Optimization of the Fermentation Medium

The influence of three carbon sources on pseudoalterin production by CF6-2 were investigated, including corn powder (0%, 1%, 3%), glucose (0%, 1%, 2%, 2.5%), and sucrose (0%, 0.1%, 0.5%, 1%). CF6-2 was cultured under the flask fermentation conditions described above in the basal medium with the carbon sources altered. The content of artery powder was optimized in the basal medium containing 0.5% sucrose. CF6-2 was cultured under the flask fermentation conditions with the concentration of artery powder changed from 0.2% to 0.8%. All treatments were carried out in triplicate.

### 4.5. Optimization of Aeration Rate and Stirring Speed in a Mini-in Parallel Fermenter System

In a mini-in parallel fermenter system with 4 fermenters (1.0 L) (Multifors, Infors HT, Bottmingen, Switzerland), a 1% (*v*/*v*) inoculum of CF6-2 was inoculated into 0.8 L of the optimized medium in each fermenter, which was cultured at 20 °C with an aeration rate of 0.5 vvm and a stirring speed of 300 rpm. After five hours of culturation, two single factors, the aeration rate (0.5 vvm, 1.0 vvm, 1.5 vvm or 2.0 vvm) and the stirring speed (100 rpm, 300 rpm, 500 rpm or 700 rpm) were investigated for their effects on pseudoalterin production. All treatments were performed in triplicate.

### 4.6. Small-Scale Fermentation

In small-scale fermentation, a 1% (*v*/*v*) inoculum of CF6-2 was inoculated into 4 L of the optimized medium in a 5-L fermenter (Bioflo & Celligen 310 fermenter, New Brunswick Scientific, Edison, NJ, USA). The temperature for fermentation was 20 °C. The aeration rate and the stirring speed were 0.5 vvm and 300 rpm, respectively, for the first five hours and were then adjusted to 1.5 vvm and 500 rpm, which were maintained until the end of fermentation. For fed-batch fermentation, 0.5 L of feeding medium was supplemented at the 9th hour, which contained 1% (*w*/*v*) artery powder, 0.2% (*w*/*v*) yeast extract, 0.33% (*w*/*v*) sucrose, 0.5 mM Na_2_HPO_4_, 0.5 mM CaCl_2_ and artificial sea water (pH 8.5). The fermentation was performed in triplicate.

### 4.7. Pilot-Scale Fermentation

In the pilot-scale fermentation, a 1% (*v*/*v*) inoculum of CF6-2 was inoculated into 40 L of the optimized medium in a 50-L fermenter (07-F42 fermenter, Shanghai Baoxing Biological Equipment Engineering Co., Ltd., Shanghai, China). The culture was fermented under the same fermentation conditions as those in the small-scale fed-batch fermentation after 3 mL of polyoxypropylene-oxyethylene polyol ether, which served as an antifoaming agent, were added. The fermentation was performed in triplicate.

### 4.8. Assay for the Effects of Sugars and Polyols on Pseudoalterin Thermostability

The effects of four sugars or polyols on pseudoalterin thermostability were investigated, including glucose (1.25 M, 1.875 M, 2.5 M), trehalose (0.625 M, 1.25 M, 1. 875 M), glycerin (6.25%, 12.5%, 25%) and sucrose (0.625 M, 1.25 M, 1.875 M). These sugars or polyols were mixed with an enzyme solution (0.6 mg/mL) at a ratio of 4:1 (*v*/*v*) prior to incubation at 37 °C. After a 30-min incubation, the residual elastase activity of pseudoalterin was measured and calculated as the percentage of the initial enzyme activity (before incubation and without sugars or polyols in the solution). The enzyme solution mixed with 50 mM Tris-HCl (pH 9.0) served as the control. Experiments were performed in triplicate.

### 4.9. Compound Stabilizer Design by Orthogonal Test

To develop an effective compound stabilizer, the concentrations of glucose, trehalose, glycerin and sucrose were further optimized by an orthogonal test. The center point values and range of the four independent variables were set on the basis of the results from the single factor experiments on these compounds (Table 1). For the combination of four variables given in Table 1, the trial was a L9 (3^4^) design with four factors and three levels, resulting in a total of nine compound stabilizers (Table 2).

The enzyme solution of pseudoalterin was mixed with each compound stabilizer at a ratio of 1:4 (*v*/*v*) and incubated at 37 °C for 6 h prior to the activity assay. The stability of the enzyme was expressed as a percentage of the residual activity compared to the initial enzyme activity (before incubation and no compound stabilizer). The enzyme solution that was incubated with 50 mM Tris-HCl (pH 9.0) served as the control. Experiments were performed in triplicate.

## Figures and Tables

**Table 1:** Factors and levels of the L9 (3^4^) orthogonal test.

Level	Factor			
	A	B	C	D
	Glucose (M)	Trehalose (M)	Glycerin (%)	Sucrose (M)
1	0.25	0.25	1.25	0.25
2	0.9375	0.625	2.5	0.125
3	1.875	1.25	6.25	0.0625

The data represent the initial concentrations of each sugar and polyol before mixing with the enzyme solution.

**Table 2:** Orthogonal test design for compound stabilizers.

Test No.	Glucose	Trehalose	Glycerin	Sucrose
1	1	1	1	1
2	1	2	2	2
3	1	3	3	3
4	2	1	2	3
5	2	2	3	1
6	2	3	1	2
7	3	1	3	2
8	3	2	1	3
9	3	3	2	1
